# Accounting for tumor heterogeneity when using CRISPR-Cas9 for cancer progression and drug sensitivity studies

**DOI:** 10.1371/journal.pone.0198790

**Published:** 2018-06-13

**Authors:** Jessica F. Olive, Yuanbo Qin, Molly J. DeCristo, Tyler Laszewski, Frances Greathouse, Sandra S. McAllister

**Affiliations:** 1 Department of Medicine, Division of Hematology, Brigham and Women’s Hospital, Boston, Massachusetts, United States of America; 2 Department of Medicine, Harvard Medical School, Boston, Massachusetts, United States of America; 3 Broad Institute of Harvard and MIT, Cambridge, Massachusetts, United States of America; 4 Harvard Stem Cell Institute, Cambridge, Massachusetts, United States of America; University of South Alabama Mitchell Cancer Institute, UNITED STATES

## Abstract

Gene editing protocols often require the use of a subcloning step to isolate successfully edited cells, the behavior of which is then compared to the aggregate parental population and/or other non-edited subclones. Here we demonstrate that the inherent functional heterogeneity present in many cell lines can render these populations inappropriate controls, resulting in erroneous interpretations of experimental findings. We describe a novel CRISPR/Cas9 protocol that incorporates a single-cell cloning step prior to gene editing, allowing for the generation of appropriately matched, functionally equivalent control and edited cell lines. As a proof of concept, we generated matched control and osteopontin-knockout Her2^+^ and Estrogen receptor-negative murine mammary carcinoma cell lines and demonstrated that the osteopontin-knockout cell lines exhibit the expected biological phenotypes, including unaffected primary tumor growth kinetics and reduced metastatic outgrowth in female FVB mice. Using these matched cell lines, we discovered that osteopontin-knockout mammary tumors were more sensitive than control tumors to chemotherapy *in vivo*. Our results demonstrate that heterogeneity must be considered during experimental design when utilizing gene editing protocols and provide a solution to account for it.

## Introduction

CRISPR/Cas9 is a useful tool that has expanded our ability to define the role of particular factors in biological processes, including cancer biology [1, 2]. Oftentimes, studies employ the CRISPR/Cas9 system to generate loss- or gain-of-function mutations in a gene of interest and then look for a corresponding phenotypic change, indicating whether or not the targeted gene is necessary and/or sufficient for a particular behavior. Widely used protocols that employ CRISPR/Cas9 to generate genetically modified cell lines often require a subcloning and/or selection step in order to isolate a particular subpopulation in which the gene of interest was efficiently edited [3–7]. In order to correctly define the role that particular factors play, for example in cancer models, it is essential to use appropriately matched controls to compare to the edited subclone(s); however, such comparisons can be complicated by the widespread heterogeneity present in tumors and cancer cell lines derived from them.

The relevance and pervasiveness of genetic and functional heterogeneity within most cancer types has become particularly appreciated over the past decade [8–10]. It is now known that even supposedly clonal cancer cell lines are composed of subpopulations with widely differing phenotypes and functional characteristics [11–13]. Genetic and phenotypic heterogeneity has also been observed in other disease models, including bacterial antibiotic resistance and in the evolution of antiviral resistance [14–17].

Due to the inherent functional heterogeneity observed in most cancer cell lines, therefore, subcloning and selection steps employed in genetic editing protocols can render the parental population an inappropriate control, as its behavior may differ from that of the selected subclonal population prior to gene editing. For example, if the aim of a study is to evaluate whether a particular gene product (protein) is relevant for primary tumor formation, it is common practice to compare the tumorigenicity of a knockout cell line with that of the parental cell line. However, if the selected subclonal population has an inherently different tumorigenic potential than the bulk parental population, it would be possible to incorrectly conclude that the knockdown of the gene of interest was responsible for any functional differences that are observed in any given biological assay.

Here we report a modified CRISPR/Cas9 targeting strategy to create appropriately matched knockout (KO) and wild-type (WT) control mammary carcinoma cell lines. We used these cell lines for both proof-of-concept and discovery studies. Our results demonstrate that it is critical to generate appropriately matched control and knockout cell lines in order to accurately evaluate the relevance of a protein of interest to cancer cell behaviors.

## Materials and methods

### Cell lines

McNeu and Met-1 murine mammary carcinoma cells (kind gifts from Drs. Michael Campbell and Johanna Joyce, respectively) were cultured as previously described [18, 19]. Briefly, cells were cultured in DMEM (Gibco) media, supplemented with 10% fetal bovine serum (FBS) and 100 U/ml penicillin-streptomycin at 37°C under 5% CO_2_. Human MDA-MB-435 cells were a generous gift from Dr. Robert Weinberg and were cultured in DMEM:F12 (1:1; Gibco), supplemented with 10% fetal bovine serum and 100 U/ml penicillin-streptomycin at 37°C under 5% CO_2_. All cell lines were validated as mycoplasma-negative. Human cells were validated using short tandem repeat (STR) profiling (Molecular Diagnostics Laboratory at Dana-Farber Cancer Institute, Boston, MA). For mouse cells, the murine strain of origin was confirmed by short tandem repeat analysis (Bioassay Methods Group, NIST).

### New gene editing protocol

Clonal subpopulations are generated from parental cell lines by sorting one single cell per well into 96-well plates using a FACSAria II cell sorter (BD Bioscience). Single cell-derived populations are subsequently allowed to proliferate for expansion. A single expanded clone is used for both control and co-transfection with the Cas9/GFP and sgRNA vectors. Select cell populations were seeded into 12-well plates overnight before transfection with 500ng pCas9_GFP and 500ng sgRNA expressing plasmids using FugeneHD (Roche). 48 hours after transfection, successfully transfected single cells are isolated by FACS sorting for GFP-positivity using a FACSAria II cell sorter (BD Bioscience) followed by recovery and expansion in 12-well plates for 2–3 days. At confluency, cells were collected for a second round of FACS sorting and single GFP-negative cells were sorted into individual wells in a 96-well plate to ensure that random Cas9/GFP integration did not occur. Following clonal expansion editing is validated using Sanger sequencing and phenotype verification is performed.

To generate luciferase/GFP-positive populations, cells were infected with lentivirus generated from pLV-Luc-IRES-GFP viral plasmids (a generous gift from Dr. Robert Weinberg’s lab) and then sorted for GFP-positive populations.

### Vector construction

The human codon-optimized Cas9 expression plasmid pCas9_GFP was a gift from Kiran Musunuru (Addgene plasmid # 44719). The sgRNA targeting mouse OPN exon 2 (5’-GTGATTTGCTTTTGCCTATT-3’) driven by human U6 promoter was synthesized at Eurofin.

### Evaluating target site modification by Sanger sequencing

OPN gene fragments were amplified with the primers OPN-F (5’-GACTTGGTGGTGATCTAGTGG-3’) and OPN-R (5’-GCCAGAATCAGTCACTTTCAC-3’) using Phire Animal Tissue Direct PCR Kit (Thermo Scientific). The resulting PCR products were then submitted for sanger sequencing (Macrogen USA).

### Animals and tumor studies

Female FVB/NJ mice 7 weeks of age were purchased from Jackson Labs (stock no. 001800). NOD/SCID mice were maintained in-house under aseptic sterile conditions. All experiments were conducted in accordance with regulations of the Children’s Hospital Institutional Animal Care and Use Committee (protocol 12-11-2308R), the MIT committee on animal care (protocol 1005-076-08), and Brigham and Women’s Hospital animal care protocol committee (2017N000056). Mice were 8–9 weeks of age at the time of study initiation. All efforts were made to minimize animal suffering. Animal facility personnel monitored the animals daily, checking for levels of food, water, and bedding in each cage. Mice were also physically checked three times a week by the investigators. The basic animal maintenance included housing the mice in cages (five per cage) with sufficient diet, water and bedding and cages were cleaned and sanitized on a regular basis. Investigators strictly adhered to approved protocols for humane endpoints; if any animal became severely ill prior to an experimental endpoint, that animal would be euthanized. Humane endpoints were defined as follows: ≥20% weight loss, rough hair coat, jaundice and/or anemia, coughing, labored breathing, nasal discharge, neurological signs (frequent seizure activity, paralysis, ataxia), prolapse, self-induced trauma, any condition interfering with eating or drinking, excessive or prolonged hyperthermia or hypothermia, tumor size ≥1.5 cm^3^ in volume. Animals were randomly assigned to treatment groups and no animals were excluded from analysis.

For tumor studies, murine mammary carcinoma cells were injected orthotopically, using a total of 10^5^ or 10^6^ McNeu cells, or 2.5 × 10^4^ or 2.5 x 10^5^ Met-1 cells implanted into the fourth mammary fat pad of 7–10 week old female FVB mice. Where indicated, either 1x10^5^ or 1x10^6^ cells of the McNeuA parental cell line were implanted subcutaneously. 2.5x10^5^ human MDA-MB-435 cells were injected subcutaneously into 8–10 week old female NOD-SCID mice. Thereafter, tumors were monitored and measured using calipers with volume calculated as 0.5(length ×width^2^).

For the Met-1 metastasis assay, mice received tail vein injections with 10^6^ cells of luciferase-labeled Met-1 cells suspended in 100 μl of sterile phosphate-buffered saline. MT-2 WT and OPN-KO clones express different levels of luciferase in vitro because they were labeled separately. Therefore, we evaluated the in vitro luciferase expression levels of these cells at the start of the experiment, prior to IV injection, and used that reading to normalize the in vivo signals. Pulmonary metastases were monitored weekly by bioluminescent imaging using the Spectrum Imaging System and Living Image software (Caliper Life Sciences, Inc.). Prior to imaging, mice were intraperitoneally administered 150 mg/kg D-luciferin (Perkin-Elmer) and were anesthetized using isoflurane inhalation. Luminescent signal was detected for the regions of interest as radiance (p/sec/cm^2^/sr) and analyzed using the Living Image Software Version 4.1 (Caliper Life Sciences). Lungs were fixed and stained using Hematoxylin/Eoisin and metastases were classified as multi- or single-focal and were counted manually on 3 separate sections spaced 50 microns apart per mouse. Total lung area was quantified using Cell Profiler and metastases counts were normalized total lung area.

### Chemotherapy

For AC-T chemotherapy trials, 2.5 × 10^5^ Met-1 Luc/GFP cells were injected into the mammary fat pad of 6–8-week-old female FVB mice. Doxorubicin (Teva), paclitaxel (Hospira), and cyclophosphamide (Sigma) were diluted in PBS for *in vivo* experiments. Mice were treated with two to four doses of 5 mg/kg doxorubicin, 10 mg/kg paclitaxel, and 120 mg/kg cyclophosphamide administered every two weeks. Doxorubicin was administered via retro-orbital injection, and paclitaxel and cyclophosphamide were administered via intraperitoneal injection.

For studies investigating the role of OPN in chemotherapeutic response, 2.5 × 10^4^ WT or OPN KO tumor cells were injected into the mammary fat pad of 6–8-week-old female FVB mice. When established tumors reached 60–80 mm^3^ in volume, treatment was initiated. Four treatment arms were included: vehicle control (PBS) on WT or OPN KO cohorts or one dose of paclitaxel (10 mg/kg), doxorubicin (5 mg/kg) and cyclophosphamide (120 mg/kg) by intraperitoneal injection (paclitaxel and cyclophosphamide) and retro-orbital injection (doxorubicin) on WT or OPN KO cohorts. Tumor growth was monitored using caliper measurements. Average tumor mass at sacrifice was measured and is presented as the average ± standard error of mean.

### Osteopontin ELISAs and western blotting

To assess circulating secreted murine osteopontin (mOPN) or human osteopontin (hOPN) protein levels, whole blood was collected in EDTA-coated tubes (VWR) and centrifuged at 1.5xg for 8 minutes to isolate plasma. mOPN and hOPN concentrations were determined by ELISA according to manufacturer’s instructions (R&D) and analyzed using a plate reader (Molecular Device).

To quantify secreted mOPN levels in conditioned medium, cells were grown to 80–90% confluence in growth medium containing 10% FBS. Then the medium was replaced with serum-free medium and was collected 24 hours later. mOPN levels in conditioned media were quantified by ELISA or western blotting.

Whole cell lysates were prepared following culture in the presence or absence of brefeldin A (used to prevent the secretion of OPN and ensure detection of protein expression). Cell lysates or concentrated conditioned medium were subjected to SDS-PAGE on 12% gels and then transferred onto a polyvinylidenedifluoride membrane, which was incubated with mouse anti-OPN (final dilution: 1:200, Clone AKm2A1, Santa Cruz Catalog # sc-21742, mouse monoclonal antibody raised against recombinant OPN of mouse origin, references with validation available on manufacturer’s datasheet) antibody at 4C overnight. After being washed, membranes were incubated with horseradish peroxidase-conjugated anti-mouse IgG for 1 hour. The enzyme bound to OPN was visualized using the SuperSignal™ West Pico Chemiluminescent kit (ThermoFisher). The blot was then stripped and incubated with rabbit anti-mouse β-actin antibody as a loading control (final dilution: 1:1000, Rockland Catalog # 600-401-886, rabbit polyclonal antibody raised against human beta-actin, references with validation available on manufacturer’s datasheet).

### Immunohistochemistry, immunofluorescence and microscopy

Formalin-fixed, paraffin embedded tissues were sectioned onto ProbeOn Plus microscope slides (Fisher Scientific) and immunohistochemistry was performed as described [20]. For immunohistochemistry studies, anti-OPN (final dilution: 1:200, Maine Biotechnology Services Catalog #MAB197P, mouse monoclonal antibody raised against recombinant OPN of human origin, [21]) or anti-e-Cadherin (final dilution: 1:100, Cell Signaling Technologies Catalog #3195T) were used and were detected using the Vector ABC kit (Vector Laboratories, Burlingame, CA, USA). For immunofluorescence, anti-OPN (final dilution: 1:50, Clone AKm2A1, Santa Cruz Catalog # sc-21742, mouse monoclonal antibody raised against recombinant OPN of mouse origin, references with validation available on manufacturer’s datasheet) was used and was detected using a goat anti-mouse IgG AF549 conjugated secondary antibody (final dilution: 1:1000, Invitrogen Catalog # A11032, polyclonal, references with validation available on manufacturer’s datasheet). Nuclei were counterstained with DAPI (Invitrogen). Images were captured with identical exposure and gain using a Nikon Eclipse N*i* microscope.

### *In vitro* chemosensitivity studies

4,000 Met-1 cells were plated in quadruplicates in 96-well plates containing growth media. The next day, vehicle (PBS) or chemotherapy (doxorubicin: .33 nM—2.2 μM; paclitaxel: 14 μM– 160 μM) was added to the plate and incubated for 72 hours. ATP levels were quantified as a surrogate measure for viability (CellTiter-Glo, Promega) using a luminometer (Perkin-Elmer).

### Statistical analyses

Data are represented as mean + SEM and analyzed by ANOVA, Student’s t-test, and/or Mann-Whitney test as indicated using GraphPad Prism 7.0, unless otherwise stated. *P* < 0.05 was considered statistically significant. Error bars represent standard deviation unless otherwise indicated.

## Results

### Selection of Her2^+^ and Estrogen receptor-negative mammary carcinoma models

We aimed to design an approach that would enable us to generate appropriately matched control and CRISPR/Cas9 knockout cell lines, while taking into account the inherent functional heterogeneity present in nearly all breast tumors and tumor-derived cancer cell lines. We hypothesized that results from studies employing standard CRISPR/Cas9 approaches, which often require a subcloning and/or selection step, would be confounded by subclonal functional heterogeneity.

As a proof of concept, we chose to study Osteopontin (OPN), a protein that we have studied previously and that is relevant for breast cancer metastasis [20, 22–28]. OPN plays an important role in metastasis and survival in many pre-clinical cancer studies, and is positively associated with metastasis as well as reduced progression-free and overall survival in breast cancer patients [27–29]. Additionally, OPN has been shown to play a role in chemoresistance in some cancer types [22, 24, 25, 30–32], but it is unclear whether this is also true of breast cancer.

Hence, we determined that the breast cancer models that we would employ must meet the following criteria: secretion of detectable levels OPN both *in vitro* and *in vivo*, capacity to form primary and metastatic tumors *in vivo*, evidence of heterogeneity, and responsiveness to chemotherapy.

Transgenic mice that specifically overexpress oncogenic proteins in the mammary fat pad are commonly employed both for the study of spontaneous breast tumors and as a source for murine breast cancer cell lines that can be allografted orthotopically in immunocompetent animals. In this study, we utilized two such murine breast cancer cell lines: McNeuA, a HER2^+^ breast cancer cell line derived from a spontaneously arising mammary carcinoma in a MMTV-*neu* transgenic mouse [19], and Met-1, an estrogen receptor-negative (ER^-^) breast cancer cell line derived from a mammary carcinoma in a MMTV-PyMT transgenic mouse (FVB/N-Tg(MMTV-PyVmT) [18].

**Fig 1:**
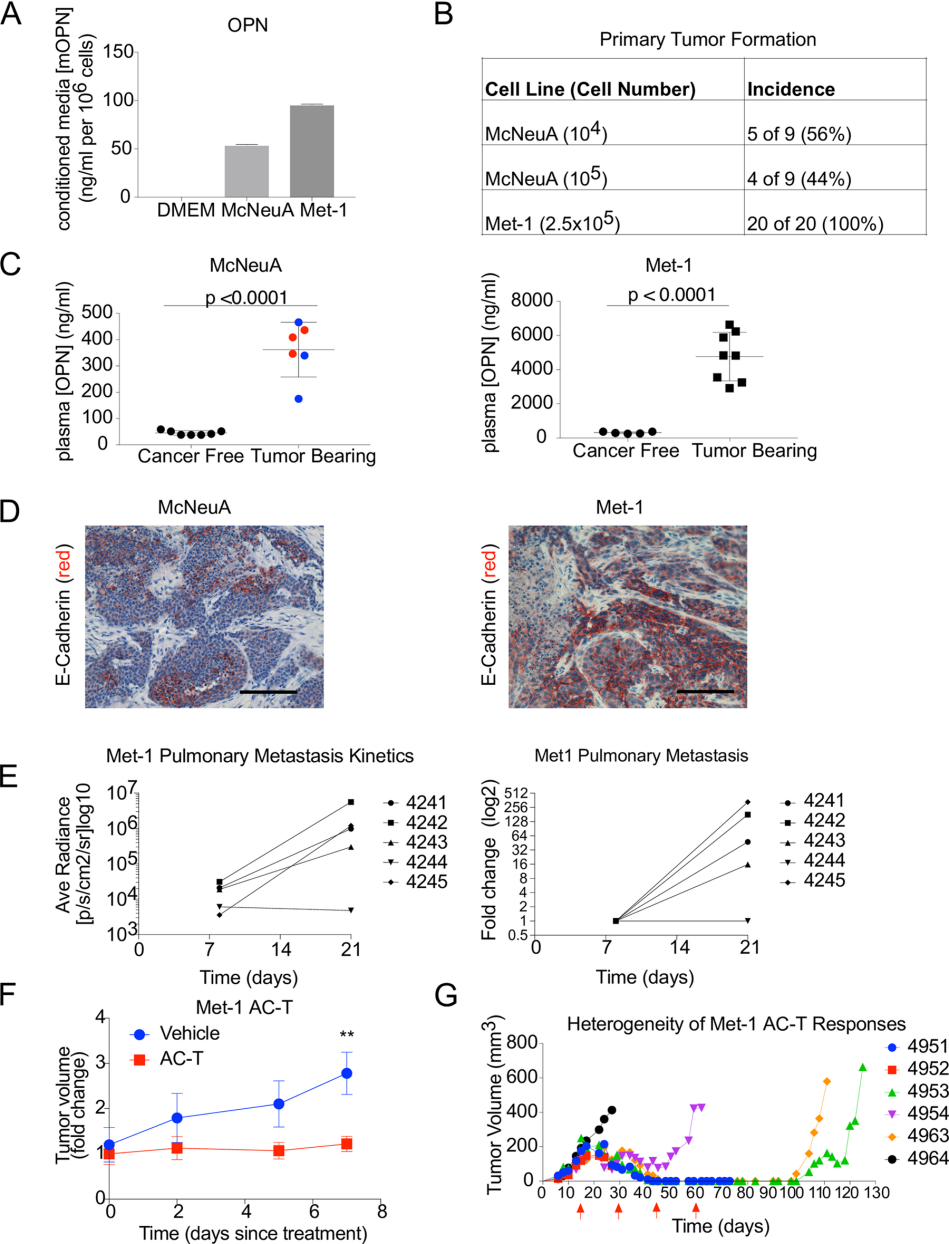
Phenotypic and functional heterogeneity of McNeuA and Met-1 breast cancer cells. **(A)** Concentration of murine OPN (mOPN; ng/ml per 10^6^ cells) in 24-hr conditioned medium of McNeuA and Met-1 murine mammary carcinoma cells represented as mean ± SD. There was no detectable mOPN in the control cell-free medium (DMEM) (2 technical replicates per group). **(B)** Incidence of tumor formation following injection of indicated numbers of McNeuA or Met-1 cells into cohorts of FVB mice. **(C)** Plasma mOPN concentration (ng/ml) in indicated cohorts of mice at experimental end points of 84 days (McNeuA) and 30 days (Met-1). For McNeuA tumor-bearing mice, blue data points represent 10,000 cells injected, red data points represent 100,000 cells injected; n = 6–7 for McNeuA cohorts; n = 5–8 for Met-1 cohorts. Error bars represent SD; statistical significance evaluated using unpaired, two-tailed Student’s t-test. **(D)** Representative images of immunohistochemical staining for murine E-cadherin (red) on recovered McNeuA and Met-1 tumors. Cell nuclei were counterstained with hematoxylin (blue). Scale bars = 100 μm. (B-D) representative of 3 independent experiments per cell line. **(E)** Average radiance (log_10_) per mouse (n = 5) as measured by bioluminescence imaging over 21-day time course following intravenous injection of 10^6^ Met-1 tumor cells into FVB mice (left graph). Fold-change (log_2_) in pulmonary metastatic burden per mouse (right graph). Representative of 2 independent experiments. **(F)** Response of orthotopic Met-1 GFP/Luc tumors to single dose combination doxorubicin (5 mg/kg), paclitaxel (10 mg/kg) and cyclophosphamide (120 mg/kg) (AC-T), n = 5–8 tumors/group. Ordinate represents time (days) following treatment. Error bars represent SEM; two-way ANOVA Sidak’s multiple comparisons test; **p<0.01. Representative of 3 independent experiments. **(G)** Growth kinetics of individual orthotopic Met-1 Luc/GFP tumors in mice injected with 2.5 x 10^5^ tumor cells at the experiment initiation, subsequently receiving 4 biweekly AC-T doses (red arrows). Numbers and colors represent individual mice.

Characterization of McNeuA and Met-1 cell lines demonstrated their potential as models for this study, as they secreted detectable levels of OPN in culture as measured by ELISA (Fig 1A). Both cell lines efficiently formed primary tumors following injection into FVB mice (Figs 1B and S1A). While both cell lines formed tumors that had an average mass of 2.3 g at the experimental end points (30 days for Met-1 and 90 days for McNeuA, or when tumors reached 1.5 mm^3^, S1B Fig), the McNeuA tumors exhibited more variability in both their tumor incidence and final tumor mass.

In both models, the tumor bearing mice had significantly elevated plasma levels of OPN relative to cancer-free cohorts whereby average OPN levels were 8-fold and 15-fold higher in the McNeuA and Met1 tumor-bearing mice, respectively, at end stage (Fig 1C). Interestingly, plasma OPN levels positively correlated with the final tumor mass in mice bearing the McNeuA tumors (S1C Fig). Immunohistochemical analysis of the recovered tumors revealed intratumoral heterogeneity for the epithelial marker E-cadherin (Fig 1D).

Previous studies have demonstrated that both of these cell lines are capable of forming lung metastases [18, 19]. We were particularly interested in the Met-1 cell line, as women with metastatic ER^-^ breast cancer most often experience pulmonary metastases [33]. We confirmed that the Met-1 cells formed pulmonary metastases, with 4 of 5 mice experiencing increased metastatic burden (~15-300-fold increases) over the experimental time course (Figs 1E and S1D).

We next tested responsiveness of Met-1 mammary carcinoma to combination doxorubicin (A), cyclophosphamide (C), and paclitaxel (T) chemotherapy (AC-T), a standard of care chemotherapy regimen for breast cancer patients with ER^-^negative disease. We first tested the sensitivity of Met-1 cells to doxorubicin and paclitaxel *in vitro* and performed an initial in vivo experiment to identify a therapeutically relevant, well-tolerated combinatorial dose. Cyclophosphamide, a pro-drug, requires activation into cytotoxic metabolites by liver enzymes in vivo and was therefore not tested *in vitro*. Treatment with both doxorubicin and paclitaxel significantly decreased viability of Met-1 cells *in vitro* (S1E and S1F Fig). *In vivo*, a neoadjuvant combination dose of doxorubicin (5 mg/kg), paclitaxel (10 mg/kg), and cyclophosphamide (120 mg/kg) was well tolerated (no weight loss; data not shown) and had a cytostatic effect on Met-1 tumor growth (Figs 1F and S1G).

To more closely emulate the clinical dosing regimen of AC-T chemotherapy, mice with Met1 mammary carcinoma were administered neoadjuvant AC-T every 2 weeks for 4 cycles. Interestingly, individual mice bearing Met-1 tumors exhibited differential responses to treatment, and in some cases, mice that initially experienced complete tumor regression eventually experienced local recurrence (Fig 1G).

Collectively, our analyses indicated that the McNeuA and Met-1 cell lines met our criteria of OPN secretion *in vitro* and *in vivo*, efficient formation of primary orthotopic tumors, and evidence of phenotypic and functional heterogeneity *in vivo*. Moreover, the Met-1 cells met the criteria of metastatic capacity and, chemosensitivity. Hence, the McNeuA and Met-1 cell lines were ideal for our investigation into the effect of tumor heterogeneity on the generation of appropriately matched control and OPN-KO cell lines.

### Heterogeneity between subclonal populations derived from McNeuA and Met-1

**Fig 2:**
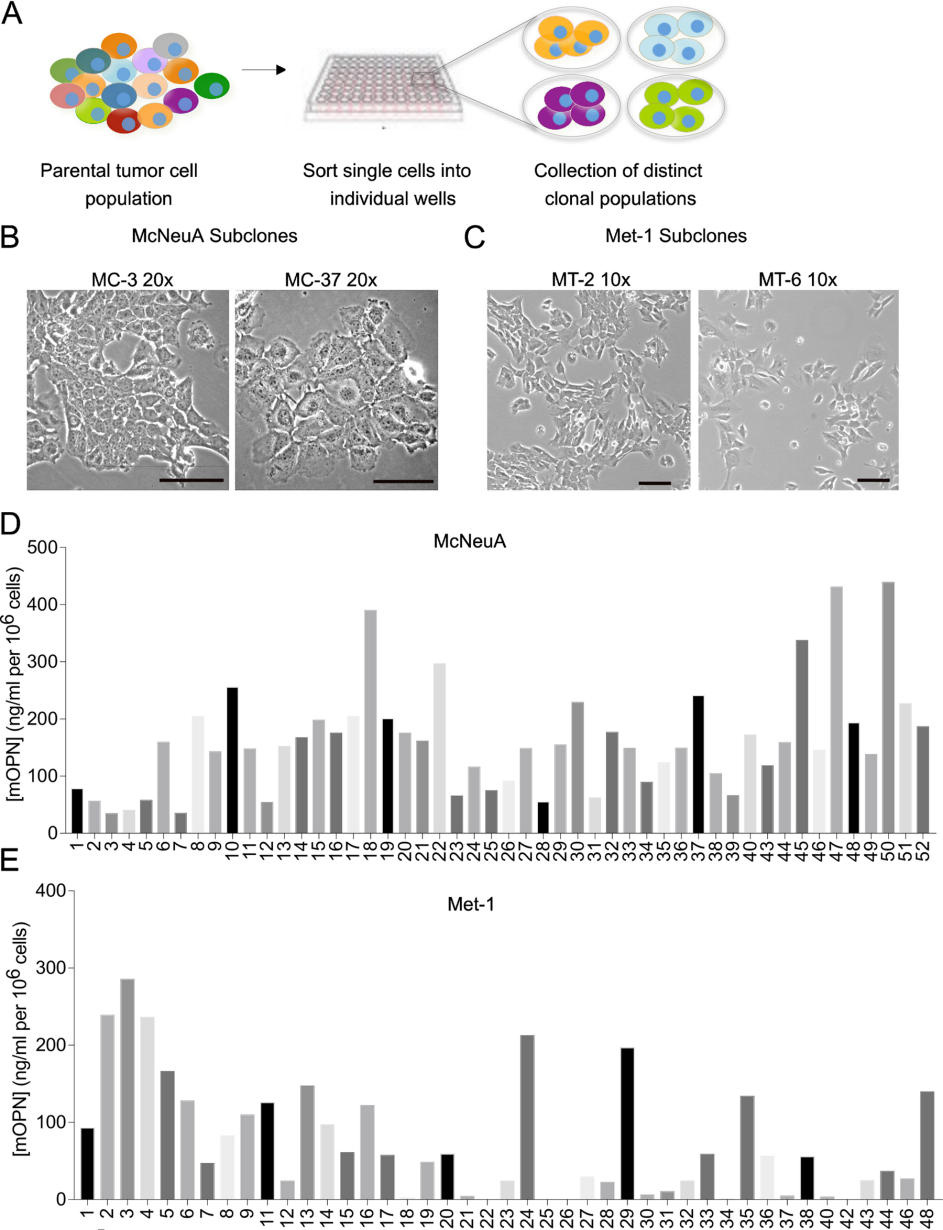
Phenotypic heterogeneity of McNeuA and Met-1 subclonal populations. **(A)** Schematic of subclone derivation from breast cancer cell lines. **(B,C)** Phase contrast images of representative McNeuA (B) and Met-1 (C) subclones to demonstrate morphologic variability. Scale bars = 100 μm. **(D,E)** Concentration of murine osteopontin (mOPN; ng/ml per 10^6^ cells) in 24-hr conditioned media from McNeuA (MC) sublcones (D) and Met-1 (MT) subclones (E).

In order to better understand whether the inherent phenotypic heterogeneity of the McNeuA and Met-1 cells lines would potentially confound the results of an OPN-knockout study, we generated single cell-derived subclonal populations from both the McNeuA (50 clones) and Met-1 (42 clones) parental cell lines (Fig 2A). The various subclonal populations exhibited morphological heterogeneity, displaying a range of epithelial and mesenchymal phenotypes in culture (Fig 2B and 2C). Cell size also appeared to vary between subclones for each given cell line (Fig 2B and 2C).

Levels of OPN secreted *in vitro* by the McNeuA and Met-1 subclones varied considerably. The McNeuA subclones secreted a range of OPN from 37.5–442.1 ng/ml per 10^6^ cells (Fig 2D), while the Met-1 subclones exhibited a range from no detectable OPN to 287.6 ng/ml per 10^6^ cells (Fig 2E). Importantly, a number of individual subclones secreted levels of OPN that differed significantly from their respective parental population. For example, OPN secretion was 6-8-fold higher in some McNeuA subclones (MC-18, MC-22, MC-45, MC-47, MC-50) and 2.5-3-fold higher in some Met-1 subclones (MT-2, MT-3, MT-4) than their respective parental populations (Figs 1A, 2D and 2E). Likewise, OPN was undetectable in some of the Met-1 cells (MT-18, MT-22, MT-25, MT-26, MT-40, MT-42) (Fig 2E). We observed similar heterogeneity of OPN secretion from clonal populations that we derived from a human melanoma cell line, MDA-MB-435 (S3A Fig), suggesting that this phenomenon is not limited to murine cell lines or breast cancer cell lines.

Taken together, these results highlighted the phenotypic heterogeneity that exists within tumor-derived breast carcinoma populations *in vitro*. We therefore explored if different clones would perform differently *in vivo* as well.

### McNeuA and Met-1 derived clonal populations behave differently *in vivo*

To understand whether various subclones that displayed different phenotypes *in vitro* would also display functional heterogeneity with respect to tumorigenesis, we injected cohorts of FVB mice orthotopically with various McNeuA or Met-1 subclonal populations and monitored tumor growth parameters over a course of 64 or 49 days, respectively. We chose to use five subclones from each cell line that secreted high levels of OPN (MC-18, MC-22, MC-45, MC-47, MC-50 and MT-2, MT-3, MT-4, MT24, MT-29) (Fig 2D and 2E). We injected either 10^5^ or 10^6^ cells of each McNeuA subclone and 2.5x10^4^ or 2.5x10^5^ cells of each Met-1 subclone.

**Fig 3:**
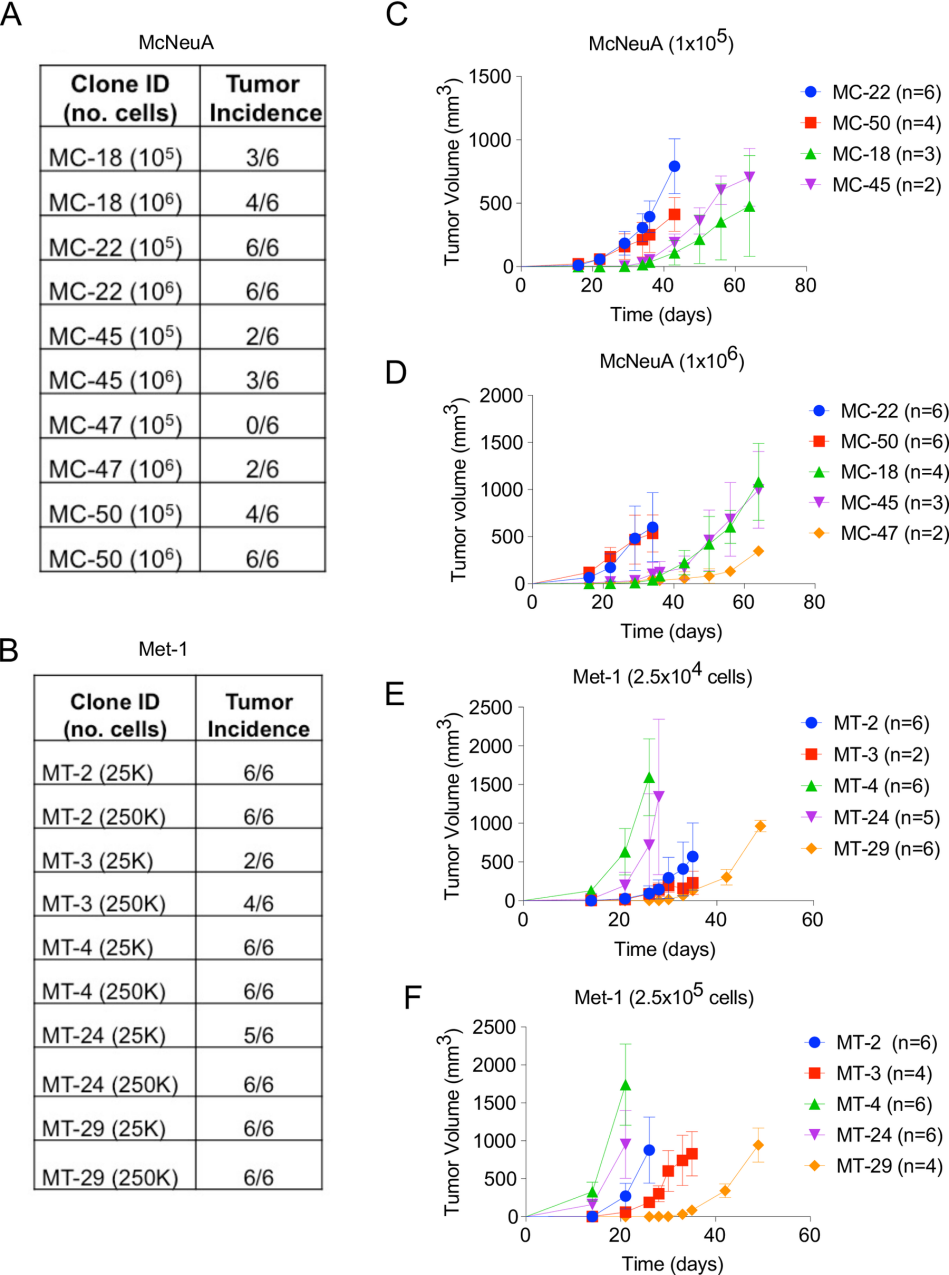
McNeuA and Met-1 subclonal populations are functionally heterogeneous in tumor incidence, latency and growth kinetics. **(A,B)** Primary tumor incidence of indicated McNeuA (10^5^ or 10^6^ cells; A) and Met-1 (2.5x10^4^ or 2.5x10^5^ cells; B) clonal populations that were injected orthotopically into FVB mice. **(C,D)** Tumor growth kinetics of indicated McNeuA clones that were orthotopically injected into FVB mice at 10^5^ (C) or 10^6^ (D) cells. Error bars represent SD; statistical significance evaluated using 2way-ANOVA. **(E,F)** Tumor growth kinetics of indicated Met-1 clones that were orthotopically injected into FVB mice at 2.5x10^4^ (E) or 2.5x10^5^ (F) cells. Error bars represent SD; statistical significance evaluated using 2way-ANOVA.

Among the McNeuA subclones, a subset of clones (MC-22 and MC-50) formed tumors with 100% incidence, while another subclone (MC-47) failed to form tumors in any mice, and incidence was only slightly higher when more cells were injected (Fig 3A). Similarly, Met-1 subclones also exhibited variable tumor incidence with 4 of 5 subclones (MT-2, MT-4, MT-24, and MT-29) forming tumors with ~100% incidence, while one subclone (MT-3) had reduced incidence to 50–66%, depending on the numbers of cells injected (Fig 3B).

Those clones that formed tumors displayed variability in latency and growth kinetics. For example, latency and growth kinetics were not statistically different between MC-22 and MC-50 when 10^6^ cells were injected (Fig 3D); however, growth kinetics differed significantly between these clones at 10^5^ (p<0.0001, Fig 3C). The subclonal populations also exhibited differences in latency. For example, when 10^6^ cells were injected, MC-22 and MC-50 had latencies of ~20 days, MC-18 and MC-45 had latencies of ~40 days, and MC-47 had a latency of ~60 days (Fig 3D).

Similarly, the growth kinetics of the Met-1 subclonal populations was also variable. When 2.5x10^4^ cells were injected, at the 28 day time point (when the MT-4 cohort had reached its endpoint), the growth kinetics of MT-4 were significantly different from the MT-2, MT-3, MT-24 and MT-29 subclones (p<0.0002, Fig 3E). The Met-1 subclones also had different latencies, with the MT-4 and MT-24 clones having shorter latencies than the other subclonal populations when either 2.5x10^4^ or 2.5x10^5^ cells were injected (Fig 3E and 3F).

The subclones derived from the human melanoma cell line also varied in incidence of subcutaneous tumor formation in NOD-SCID mice, with some clones (i.e. 11, 28, 29, 30) unable to form tumors *in vivo* (S2B Fig). Moreover, tumor mass at the experimental end point varied considerably among these subclones (S2B Fig).

Critically, a number of individual subclonal populations from each tumor model exhibited different tumor formation capabilities than the respective bulk parental population from which they were derived. For example, while the parental Met-1 tumor cell line formed orthotopic tumors with 100% incidence, the MT-3 subclonal cell line formed tumors with only 60% incidence when the same number of cells was injected (Figs 1B and 3B). This was also true of the human xenograft model (S2B Fig).

These observations revealed the considerable subclonal heterogeneity that exists within human carcinoma and murine mammary carcinoma cell lines and that the behavior of individual subclones differs from their respective parental populations.

### Evidence that identification of proper controls is necessary for correct interpretation of experimental findings

Traditional CRISPR/Cas9 editing protocols begin with infection or transfection of the bulk parental population [3–7]. For this reason, the unedited or mock-infected parental cell line is typically used as a control. Due to the inefficiency of infection and/or editing in certain cell lines (especially tumor cell lines that are hyperploid), there is often a subclonal selection step that follows the initial infection and then a validated, edited subclone is used for subsequent experimentation. Our initial characterizations of the McNeuA and Met-1 parental and subclonal populations demonstrate why one must use caution when considering this commonly used approach.

In some scenarios, subclonal heterogeneity could confound interpretation of knockout efficiency. For example, 23% of the Met-1 subclones have low or no detectable secreted OPN (Fig 2E). Hence, if one randomly selected one of these clones (e.g. MT-42) and evaluated the functional success of the OPN KO by comparing its OPN secretion levels to that of the parental Met-1 cell line, a failed knockout attempt or false positive result could be overlooked.

In another scenario, if the clonal population that was selected after CRISPR/Cas9 OPN-knockout happened to be clone MT-3 and its orthotopic tumor penetrance was compared to that of the parental Met-1 population, then one could erroneously interpret the necessity of OPN for primary tumor formation, when in fact this clone, prior to OPN knockout, already inherently forms tumors with lower incidence (~66%) than the parental population (100%) (Figs 1B and 3B).

Likewise, comparing two subclonal populations, even those that secrete similar levels of OPN and form tumors with the same incidence, could also lead to spurious results. For example, if one randomly selected MT-29 as an OPN KO clone and MT-4 as a control, then incorrect conclusions could be drawn about the role of OPN in tumor growth. This is because prior to OPN KO, both clones express similar levels of OPN (~225 ng/ml; Fig 2E) and form tumors with similar incidence (Fig 3B) but MT-29 inherently exhibits significantly longer latency and reduced growth kinetics than MT-4 (Fig 3E and 3F). The same holds true for MC-18 and MC-50, which secrete similar levels of OPN (~400 ng/ml; Fig 2D), but incidence of tumor formation after injecting 10^6^ cells is ~17% for MC-18 and 100% for MC-50 (Fig 3A). Hence, the chances of randomly selecting functionally equivalent clones–such as MC-22 and MC-50, which secrete similar levels of OPN (>250 ng/ml; Fig 2D), form tumors with similar incidence (100%; Fig 3A), and display similar growth kinetics (Fig 3B)–are low without extensive characterization of individual clones prior to gene editing.

Our results provided evidence that neither the parental population nor other subclones would represent an appropriately matched wild-type control for a CRISPR/Cas9 knockout cell line that was selected after the gene editing step. The only appropriate control would be to compare the behavior of edited and unedited cells derived from the same clonal population. We therefore concluded that a modified strategy should be developed to account for heterogeneity and enable the generation of appropriately matched cell lines.

### Generating *spp1* knockout clonal populations via CRISPR/Cas9

**Fig 4:**
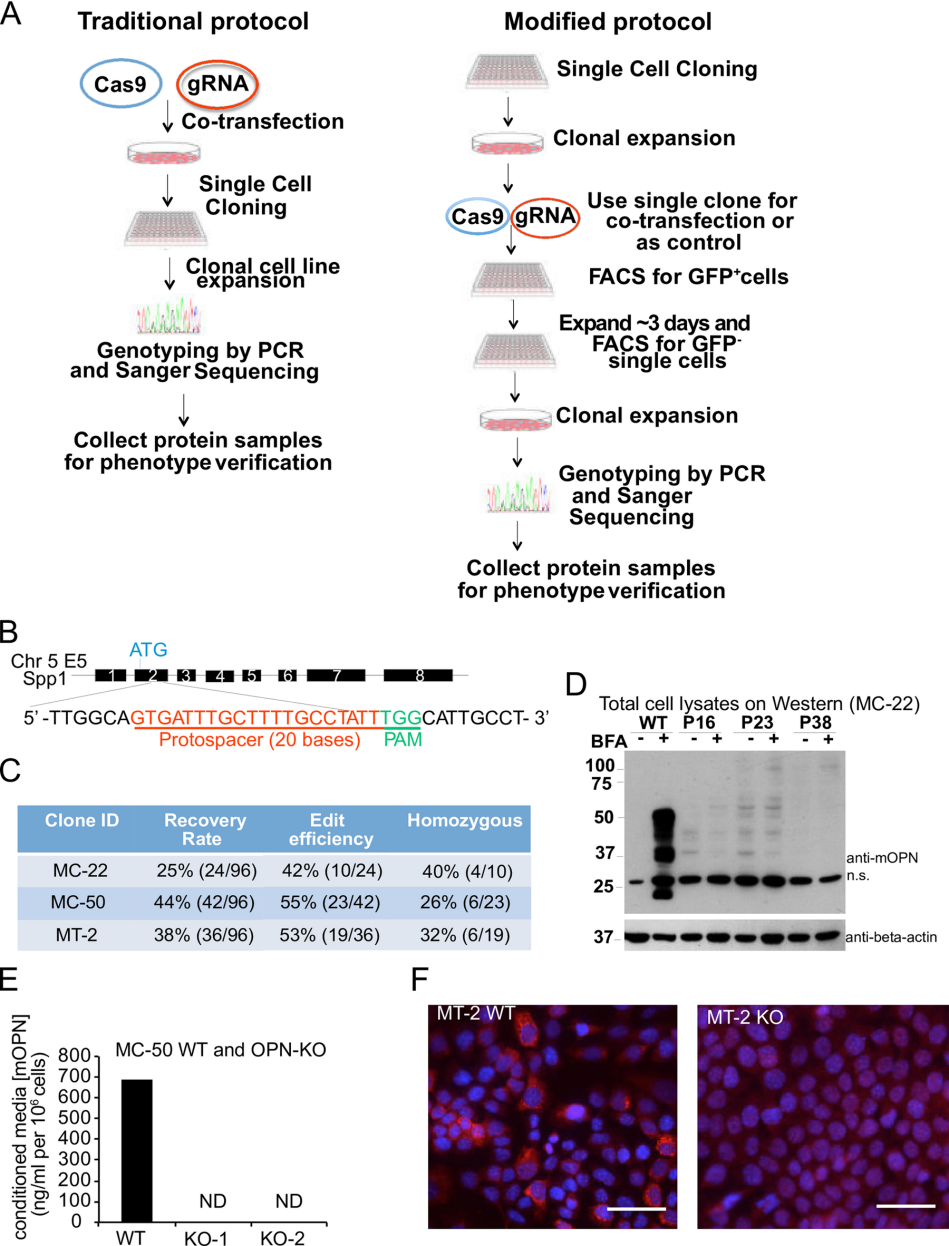
Generation of appropriately matched wild-type and OPN knockout cell lines using CRISPR-Cas9 mediated gene editing. **(A)** Schematic of traditional and modified CRISPR/Cas9 based gene editing protocols. **(B)** Schematic diagram of sgRNA targeting the *spp1* gene loci. Protospacer sequence is highlighted in red. Protospacer adjacent motif (PAM) sequences are presented in green. **(C)** Recovery rates, gene editing efficiency, and rate of homozygous targeting of the OPN gene in indicated subclones. **(D)** Western blot for OPN protein in MC-22 WT and edited clones (P16, P23, and P38) cultured in the presence or absence of brefeldin A (BFA). Expected multiple Osteopontin isoforms were detected between ~37–50 kD. A non-specific band was detected in each sample, indicated by “n.s”. **(E)** Concentration of murine osteopontin (mOPN) in 24-hr conditioned media from MC-50 WT and edited clones (MC-50-KO1 and MC-50-KO2). mOPN levels were normalized to final cell count. Osteopontin was undetected (ND) in conditioned media collected from both edited subclones. **(F)** Immunofluorescence cytochemical staining for mOPN (red) in MT-2 WT and a validated MT-2 OPN-KO clone. Nuclei are counterstained with hematoxylin (blue). Scale = 100 μm.

One would not have known *a priori* about differences in subclonal biological phenotypes and experimental outcomes by taking traditional approaches to gene editing. Therefore, we developed a modified CRISPR-Cas9 editing protocol for generating matched control and knockout cells. Appropriate subclonal populations that we had generated and characterized were chosen for CRISPR/Cas9 gene targeting based on the desired biological properties of high intrinsic levels of OPN secretion and orthotopic tumor incidence of 100%. We identified three clonal populations that fit these criteria: MC-22, MC-50, and MT-2 (Figs 2D, 2E, 3A and 3B). In contrast to traditional CRISPR/Cas9 protocols, we used single cell-derived subclonal populations that we had generated prior to CRISPR/Cas9 gene targeting (Fig 4A).

We used our modified CRISPR/Cas9 editing strategy to delete the *spp1* gene (which encodes Osteopontin) in each subclonal population in order to generate OPN KO cell lines. To do so, the individual subclonal populations were transiently co-transfected with a human codon-optimized spCas9-2A-GFP fusion protein expression plasmid (Addgene plasmid #44719) and a plasmid harboring a sgRNA targeting exon 2 of *spp1* (Fig 4B). After 24 hours, the GFP-positive (and therefore successfully transfected) Cas9-expressing cells from each subclonal population were collected by FACS and allowed to expand in culture for at least six doublings (~3 days) (Fig 4A). By giving transfected cells more time to recover from FACS sorting, we observed improved single cell cloning recovery rates for the MC-22, MC-50, and MT-2 subclones (respectively 42%, 55%, and 53%, Fig 4C) compared to transfected cells that were directly sorted as single cells, in which the recovery rate was ~5% in an initial trial (data not shown). The higher colony recovery rate and enrichment of Cas9 expressing cells during the first sorting step allowed us to achieve both higher editing efficiency and more homozygously edited clones (Figs 4C and S3).

Due to the transient nature of our transfection protocol, only cells in which the Cas9-GFP fusion protein had been randomly integrated would maintain GFP expression past this point. In order to avoid random integration of the Cas9 expression plasmid into the genome, a second round of single-cell sorting by FACS was employed to isolate cells that had not undergone a Cas9 integration event by sorting and selecting for GFP-negative cells (Fig 4A). Single cell-derived subclones were then expanded in culture.

We next employed Sanger sequencing to identify the edited subpopulations from among the recovered subclones (S3A Fig). Of the recovered subclones from the MC-22, MC-50, and MT-2 lines, a subset of the single cell clones contained either a hemizygous or homozygous mutation in the *spp1* gene, representing editing efficiencies of 42%, 55%, and 53%, respectively (Fig 4C). We found that MC-50 clone is hyperploid for the chromosome region containing *spp1* based on partially edited clones’ sequencing result (S3B Fig) and this observation was further validated by genotyping these clones using TA cloning and Sanger sequencing (data not shown). Between 26–40% of the successfully edited clones contained homozygous mutations (Fig 4C).

We validated loss of OPN protein expression in each of the OPN KO clones compared to its appropriately matched control using western blotting, ELISA of conditioned media, or immunocytochemistry. We observed no detectable OPN protein (Fig 4D–4F), demonstrating that our CRISPR/Cas9 editing strategy was successful and we had generated authentic OPN KO subclonal cell lines.

### Osteopontin is dispensable for primary tumor growth

Most studies, including our own, report that OPN is dispensable for primary tumor growth, but is critical for metastasis due to its effects on tumor cells, the host systemic environment, and the tumor microenvironment [20, 23, 26]. Therefore, successful generation of appropriately matched KO and WT cell lines should also reflect these properties (e.g., loss of OPN should have no effect on primary tumor growth, but should alter metastatic ability). This makes OPN an ideal protein to test our concept because its dispensability for primary tumor growth means that WT and OPN KO clones should exhibit similar primary tumor growth kinetics and incidence. Therefore, we tested the tumor formation capabilities of the matched clones.

**Fig 5:**
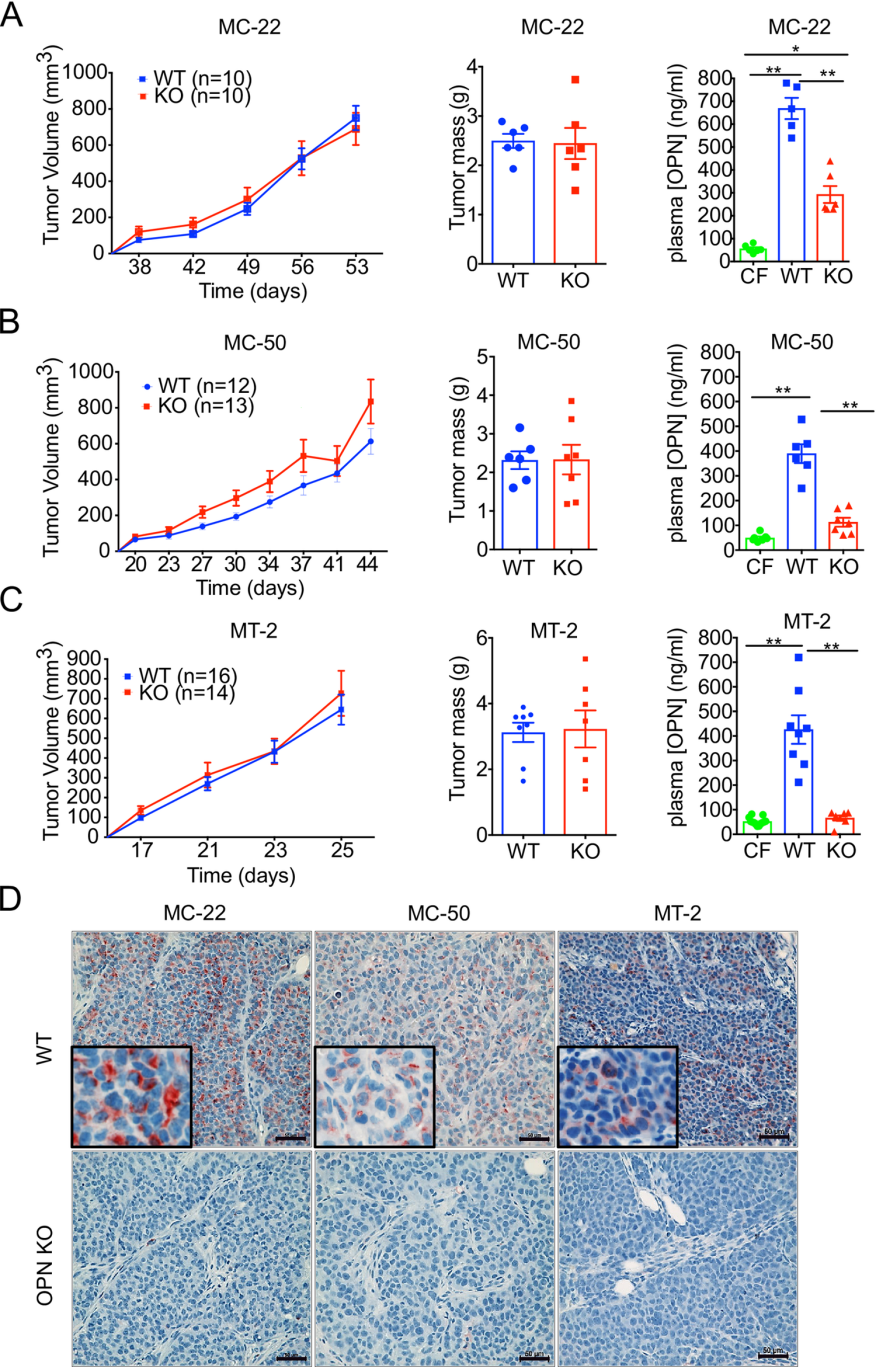
OPN depletion does not affect primary tumor formation in murine models of HER2^+^ and ER^-^ breast cancer. **(A-C)** FVB mice were orthotopically injected with 10^5^ MC-22 (A), 10^5^ MC-50 (B), or 2.5 x 10^4^ MT-2 (C) cells. Growth kinetics (mm^3^) of orthotopic tumors of WT (blue lines) and validated OPN-KO clones (red lines). Mass of primary tumors from WT (blue) or OPN-KO (red) cohorts at experimental end points. No statistically significant differences were determined by 2way ANOVA (tumor growth kinetics) or unpaired, two-tailed Students’ t-test (tumor mass) statistical analyses. Circulating plasma murine osteopontin (mOPN) levels from cancer-free (green) or tumor bearing mice from the MC-22, MC-50, or MT-2 WT (blue) or OPN-KO (red) cohorts (One-way ANOVA: *** p = 0.0003, **** p < 0.0001). Error bars represent SD. **(D)** Representative immunohistochemical staining for mOPN (red) in tumors derived from MC-22, MC-50 and MT-2 WT and validated OPN-KO cell lines. Cell nuclei counterstained with hematoxylin (blue). Scale bar = 50 μm.

WT and OPN KO MC-50 cells (2x10^5^), MC-22 cells (1x10^5^) or MT-2 cells (2.5x10^4^) were orthotopically injected into FVB mice and were allowed to grow until tumors reached ~1 cm^3^. Loss of tumor-derived OPN did not significantly affect growth kinetics or the final mass of any of the tumors derived from matched subclonal cell lines (Fig 5A–5C). In fact, there were no significant differences in any other tumor growth parameters (Fig 5A–5C) or spleen mass (S4A Fig) between cohorts bearing WT and the respective matched OPN KO tumors.

As a control, we tested the concentration of circulating plasma mOPN in the tumor-bearing mice and cancer-free controls. As expected, mOPN plasma levels were elevated in the mice bearing WT tumors relative to the cancer-free cohort, and plasma OPN levels were significantly reduced in the mice bearing KO tumors relative to WT (Fig 5A–5C). Plasma OPN levels from the cohorts of mice bearing MC-50 and MT-2 OPN KO tumors were not significantly different from their respective cancer-free cohorts (Fig 5A–5C). However, plasma OPN from mice bearing MC-22 KO tumors was significantly higher than the cancer free controls (Fig 5A), suggesting that clone MC-22 may in fact induce an elevation in host-derived OPN.

It is important to note that if we had used the parental McNeuA cell line as a WT control rather than the appropriately matched WT MC-22 cell line, we would have failed to see a significant difference in the circulating OPN levels between cohorts (S4B Fig). This observation would not have been possible using a traditional CRISPR/Cas9 gene editing protocol, once again highlighting the strength of our system and the necessity of using appropriately matched control cell lines in knockout studies.

Finally, we visualized OPN expression in the tumors that formed in each cohort using immunohistochemical staining. We observed positive staining for OPN in the WT MC-22, MC-50, and MT-2 tumors, but did not detect any OPN^+^ cells in the corresponding OPN KO tumors (Fig 5D), confirming that the OPN KO was successful. These observations provided further evidence that any circulating OPN detected in mice injected with the OPN-KO clones (Fig 5A–5C) was host-derived rather than tumor derived.

Together, these results demonstrated that our modified CRISPR/Cas9 gene editing protocol can be successfully used for studies examining the role of a gene in primary tumor outgrowth.

### Loss of osteopontin reduces multifocal metastatic outgrowth

Osteopontin is considered a biomarker for tumor progression and is detected at higher levels in more aggressive tumors than their low-grade counterparts, is elevated in the serum of patients with metastatic disease, and is included in gene lists predicting poor prognosis for many cancer types [28, 34–40]. Although OPN is most often dispensable for primary tumor growth, OPN is necessary for metastasis [20, 41–43].

**Fig 6:**
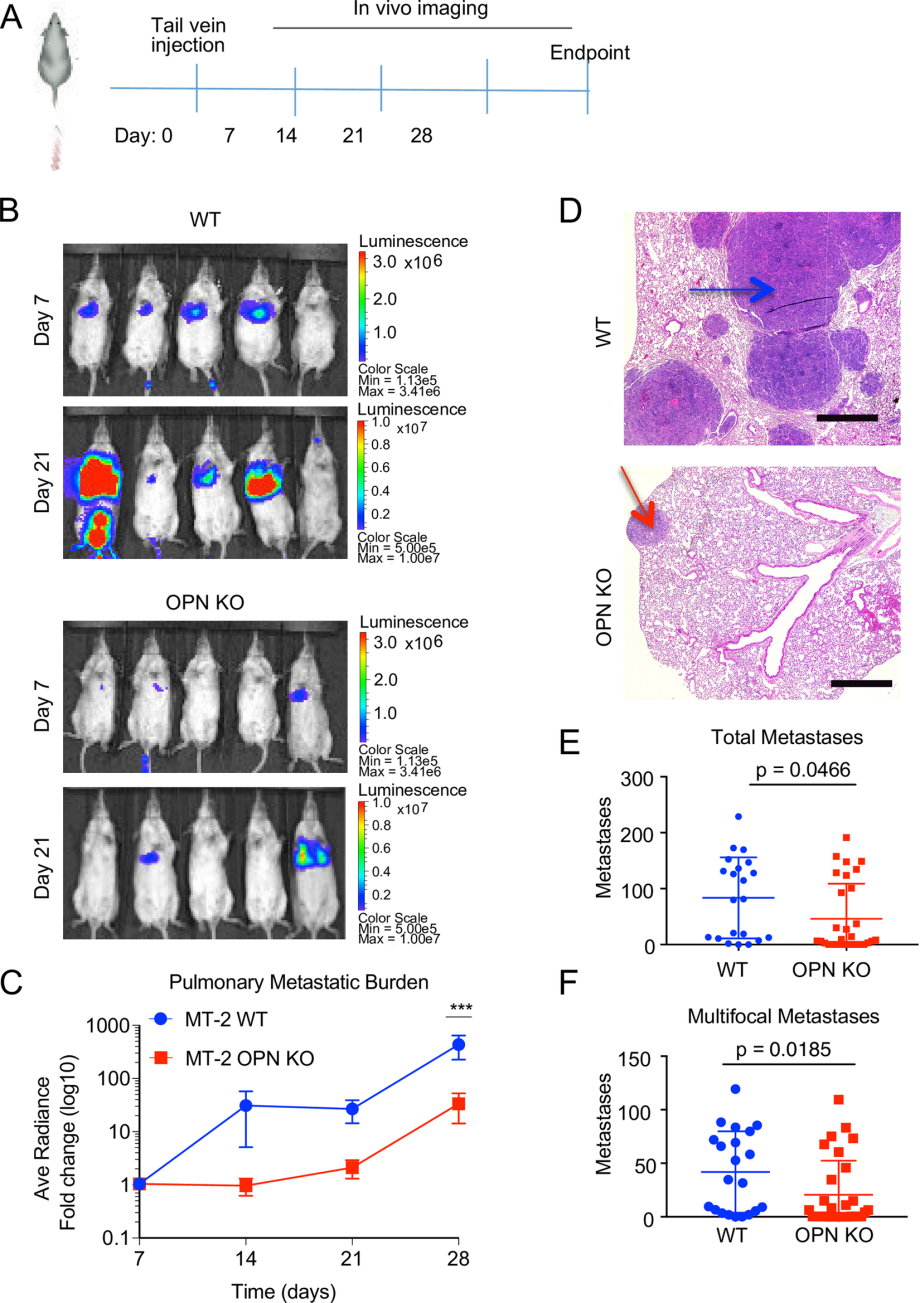
Matched wild type and knockout OPN cell lines can be used for pre-clinical metastasis studies. **(A)** Experimental schema for metastasis assay. **(B)** Representative *in vivo* bioluminescent images of mice injected with MT-2 WT or MT-2 OPN KO after 7d and 21d. **(C)** Average fold change of bioluminescent signal (radiance (p/sec/cm^2^/sr), log10, normalized for differences in Luciferase expression between cell lines) from mice with MT-2 WT (blue) or MT-2 OPN KO (red) at indicated time points. (unpaired, two tailed t-test: *** p = 0.000067). Error bars represent SEM. **(D)** Representative hematoxylin & eosin staining of lungs from mice that received tail vein injections of MT-2 WT or MT-2 OPN KO cells. An example of a multifocal metastasis is marked with a blue arrow and an example of a single focus metastasis is marked with a red arrow. Scale = 1000 μm. **(E)** Quantification of total metastases in MT-2 WT (blue) and MT-2 OPN KO (red) cohorts (WT n = 21, KO n = 30; Mann-Whitney, p = 0.0466). Error bars represent SD. **(F)** Quantification of multifocal metastases in MT-2 WT (blue) and MT-2 OPN KO (red) cohorts (WT n = 21, KO n = 30; Mann-Whitney, p = 0.0185). Error bars represent SD.

Met-1 cells are highly metastatic [18] (Fig 1E) and therefore serve as an ideal pre-clinical model of ER-negative disease to test whether our CRISPR/Cas9 system is useful for metastasis studies. To address this question, we labeled the MT-2 WT and MT-2 OPN KO cell lines with a dual GFP/luciferase reporter and injected the labeled cells intravenously via the tail vein into cohorts of mice (Fig 6A). Metastasis formation was monitored using bioluminescent *in vivo* imaging at weekly intervals.

Metastatic burden was decreased in the MT-2 OPN KO cohort relative to that of the MT-2 WT cohort, as indicated by the marked reduction in the fold change of bioluminescent signal in the MT-2 OPN KO cohort at day 28 (p = 0.000067 at day 28, p>0.05 for all other time points; Fig 6B and 6C). As further confirmation, we analyzed H&E lung sections at the experimental end point and quantified the numbers of single and multifocal metastases. There were significantly fewer total and multifocal pulmonary metastases in mice that had been injected with the OPN KO cells compared to mice that had been injected with OPN WT cells (Fig 6D–6F). Additionally, the average number of single-focus metastatic outgrowths was also reduced in mice in the OPN KO cohort compared to the WT cohort (S5 Fig).

Collectively, our results established that by using appropriately matched cells, we could confidently conclude that OPN is necessary for metastatic colonization and that our CRISPR/Cas9 protocol is useful for pre-clinical metastasis studies.

### Loss of osteopontin enhances chemosensitivity

Resistance to standard chemotherapies remains a significant clinical problem, particularly for triple-negative breast cancer [44]. In order to interrogate whether OPN contributes to chemoresistance in breast cancer models, we tested the MT-2 WT and KO cell lines for sensitivity to AC-T chemotherapy in vivo.

**Fig 7:**
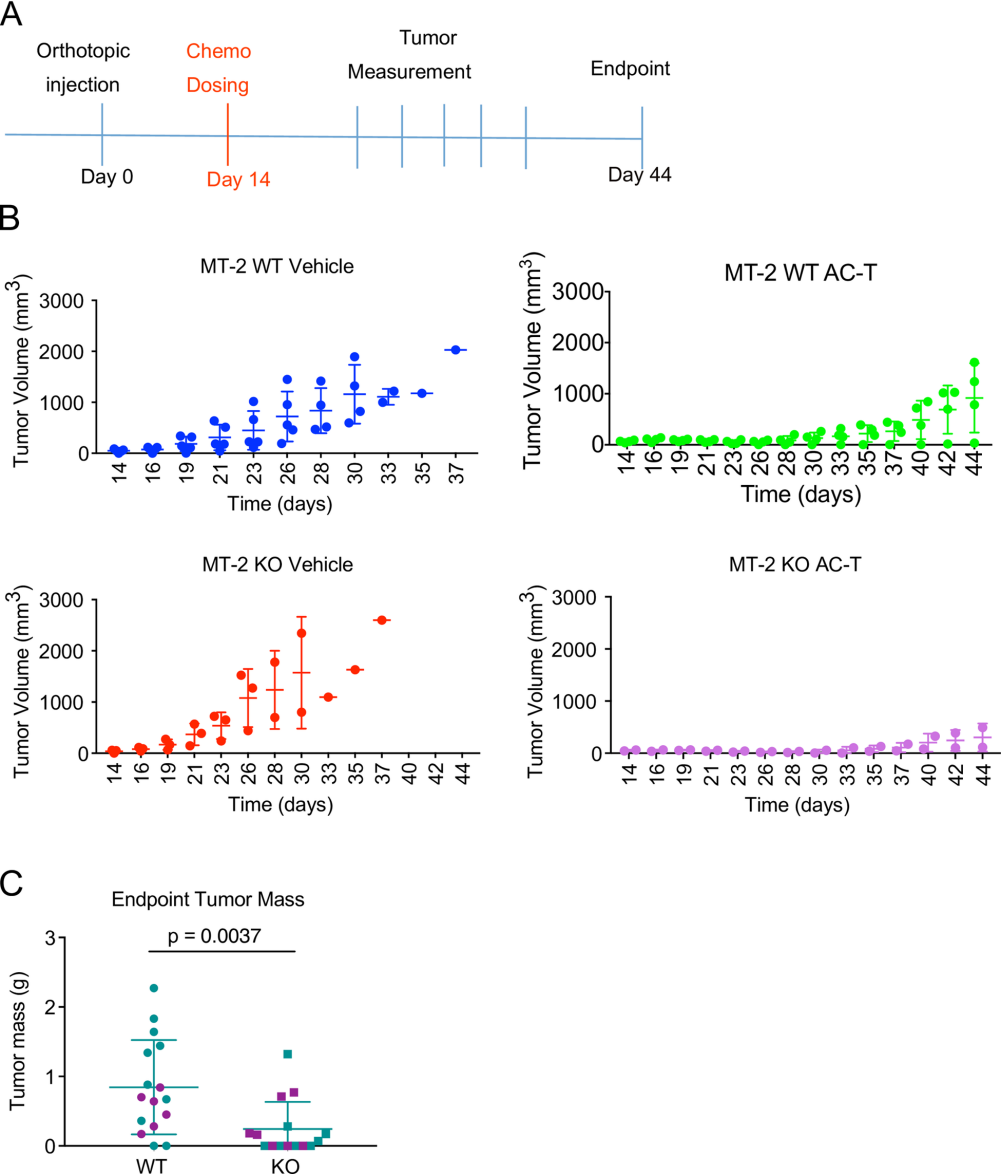
MT-2 OPN-KO derived tumors exhibit enhanced chemosensitivity *in vivo*. **(A)** Experimental schema. 2.5 × 10^4^ MT-2 WT or OPN-KO tumor cells were injected into the mammary fat pads of 6–8-week-old female FVB mice. A single dose of AC-T was initiated at 14 days, when tumors reached ~60–80 mm^3^ in volume, and tumor growth was monitored periodically until the end point of 44 days. Error bars represent SD. **(B)** Tumor growth kinetics for MT-2 WT vehicle (blue; n = 5) and AC-T treated (green; n = 4) and MT-2 OPN-KO vehicle (red; n = 3) and AC-T treated mice (purple; n = 2). Representative of 3 biological repetitions. Error bars represent SD. **(C)** Endpoint tumor mass for MT-2 WT and MT-2 OPN-KO AC-T treated mice from 2 separate experiments (Mann-Whitney, p = 0.0037; endpoint tumor mass was not measured during the first of the three experimental repetitions). Data points from individual repetitions are represented with different colors. Error bars represent SD.

We injected 2.5 × 10^4^ MT-2 WT or matched OPN KO tumor cells into the mammary fat pads of FVB mice. When established tumors reached ~60–80 mm^3^ in volume (14 days), animals were randomized based on tumor volume and enrolled into either vehicle control (PBS) or AC-T chemotherapy treatment cohorts (Fig 7A).

MT-2 WT and MT-2 KO tumors exhibited sensitivity to AC-T treatment relative to their respective vehicle-treated cohorts (Fig 7B). However, in response to AC-T, the MT-2 KO tumors exhibited reduced growth kinetics compared to their MT-2 WT counterparts in three independent trials (Fig 7B). Likewise, final tumor mass was significantly lower in the MT-2 KO treatment cohorts compared to the MT-2 WT treatment cohorts (Fig 7C). Sensitivity to doxorubicin and paclitaxel was not apparent *in vitro* (S6A and S6B Fig). Hence, the enhanced sensitivity observed *in vivo* could be due to the effects of OPN only on cyclophosphamide resistance, the host microenvironment, or both.

Together, these data established that elimination of OPN expression enhances chemosensitivity of the MT-2 breast cancer population.

## Discussion

The ability to genetically edit a cell line to either suppress, knockdown, induce, overexpress, knock-in, or mutate a protein of interest provides an indispensible tool for biological research. However, our work demonstrates that studies designed to test necessity or sufficiency of genes/gene products without choosing appropriately matched unedited controls run the risk of detecting false positive or false negative results due to inherent phenotypic differences in subclonal cellular populations that result from heterogeneity. Our alternative approach to generate subclones and screen for desired phenotypes prior to genetic manipulation provides one solution to this problem.

As we demonstrated through proof-of-concept studies, our approach works well for hypothesis-testing experimentation, when biological phenotypes to be tested are defined. Another benefit to our modified approach is that characterization of subclone phenotypes may enable one to select a range of biological properties that could be tested. Moreover, this approach enables discovery of novel properties for which mechanistic insight could be obtained in a straightforward manner. For example, one of our subclones (MC-22 KO) stimulated elevated host plasma OPN while another clone (MC-50 KO) did not, thereby enabling one to compare properties (e.g., gene expression) of related clones to yield mechanistic insights. While our approach takes added time and expense, it ensures that the real function of a specific protein of interest is uncovered during experimentation.

One caveat of our approach is that isolating particular subclonal populations removes the inherent heterogeneity of a cell line, which could have important biological consequences. This is particularly relevant in circumstances in which the biology is not well understood. If heterogeneity is desirable, then one could employ a clonal pooling approach, thus ensuring that a given experiment is both properly controlled and that the heterogeneous nature of the parental cell line is not lost.

It has been reported that functional heterogeneity can arise even within a ‘clonal’ cellular population as a result of cell plasticity or epigenetic alteration [13]. Hence, although we did not test clonal plasticity in our system, it is reasonable to hypothesize that a high degree of cellular plasticity could cause differences between matched control and edited populations that may not be due to the target gene. Limiting the *in vitro* passage of the cell lines to minimize chances for additional selection and monitoring for unexpected functional changes in control cell lines may help to prevent this added complication.

Our new experimental approach led us to discover an important function of OPN in resistance to a standard breast cancer chemotherapy regimen. Use of our matched wild type and OPN-deficient subclones will enable future studies to determine the mechanism through which OPN acts to promote this chemoresistance in breast cancer. It appears that it may rely on a non-cell intrinsic mechanism, as the reduced chemosensitivity was only observed *in vivo* and not *in vitro*. The matched subclones we report here provide a valuable tool to expand such studies.

## Supporting information

S1 Fig Met-1 and McNeuA parental tumor characteristics. **(A)** Individual and average tumor growth kinetic rates from FVB mice orthotopically injected with 2.5x10^5^ Met-1 cells. Error bars represent SD. **(B)** Endpoint tumor masses of mice injected with 10^5^ (red) or 10^6^ (blue) McNeuA cells or 2.5x10^5^ Met-1 cells. Error bars represent SD. **(C)** Circulating plasma osteopontin (OPN) levels were measured using ELISA and were plotted against the primary tumor mass in the corresponding animal. **(D)** Representative hematoxylin & eosin staining of lung tissue from a mouse that received intravenous injection of Met-1 cells. An example of a pulmonary metastasis is marked with a blue arrow. Scale = 1000 μm. Representative of two independent experiments. **(E,F)** Viability of Met1 GFP Luc cells treated in vitro with various doses of doxorubicin and paclitaxel for 72 hours. Representative of three independent experiments. Error bars represent SEM. **(G)** Tumor growth kinetics of the Met-1 Luc/GFP parental cells injected orthotopically into FVB mice at 2.5 x 10^5^ cells treated with two bi-weekly doses of either vehicle (blue, n = 6) or AC-T (red, n = 8). Error bars represent SEM.
(TIF)

S2 Fig MDA-MB-435 subclonal populations are heterogeneous. **(A)** Human osteopontin (hOPN) secreted into culture medium by MDA-MB-435 parental cells (P1-3) and single cell clones after 24h, normalized for the number of cells in each well (n = 3 replicates per cell line). **(B)** Average mass (mg) of tumors 60 days after subcutaneous injection of 2.5x10^5^ MDA-MB-435 parental cells (P1-4) or indicated subclones into NOD-SCID (n = 5 mice per cohort).
(TIF)

S3 Fig Sanger sequencing of matched wild type and CRISPR-Cas9 OPN knockout cell lines. **(A)** Examples of coding-frame shift confirmed to be homozygous in MT-2, MC-22 and MC-50 clones by Sanger sequencing as reported in Fig 4C**. (B)** Example of coding-frame shift confirmed to be heterozygous as reported in Fig 4C.
(TIF)

S4 Fig OPN depletion does not affect final primary tumor mass or spleen mass in murine models of HER2^+^ and ER^-^ breast cancer. **(A)** Final spleen mass was measured in mice injected with either MC-22, MC-50, or MT-2 WT or OPN-KO cell lines. No significant difference was observed between WT and KO cohorts for each clone (unpaired, two-tailed Student’s t-test). **(B)** Circulating plasma mOPN levels were measured from mice bearing either McNeuA Parental or MC-22 OPN-KO primary tumors using ELISA (unpaired, two-tailed t-test, p = 0.2480). Error bars represent SD.
(TIF)

S5 Fig OPN knockout results in reduced metastatic burden. Quantification of single focus metastases in MT-2 WT (blue) and MT-2 OPN KO (red) cohorts (WT n = 21, KO n = 30; Mann-Whitney, p = 0.1248). Error bars represent SD.
(TIF)

S6 Fig Enhanced chemosensitivity of OPN-depleted cell lines to doxorubicin and paclitaxel is not observed *in vitro*. (A,B) MT-2 WT or MT-2 OPN-KO cells were plated in quadruplicate and were treated with various doses of doxorubicin (A) or paclitaxel (B) 24 hours after plating. ATP levels were quantified 72 hours after treatment as a surrogate measure for viability using Cell-Titer Glo and were normalized to vehicle treated. Error bars represent SD.
(TIF)
