# Supplementary figures and images for: Accounting for tumor heterogeneity when using CRISPR-Cas9 for cancer progression and drug sensitivity studies
Source: PLoS One. 2018 Jun 13;13(6):e0198790. doi: 10.1371/journal.pone.0198790 (PMC5999218; doi:10.1371/journal.pone.0198790)

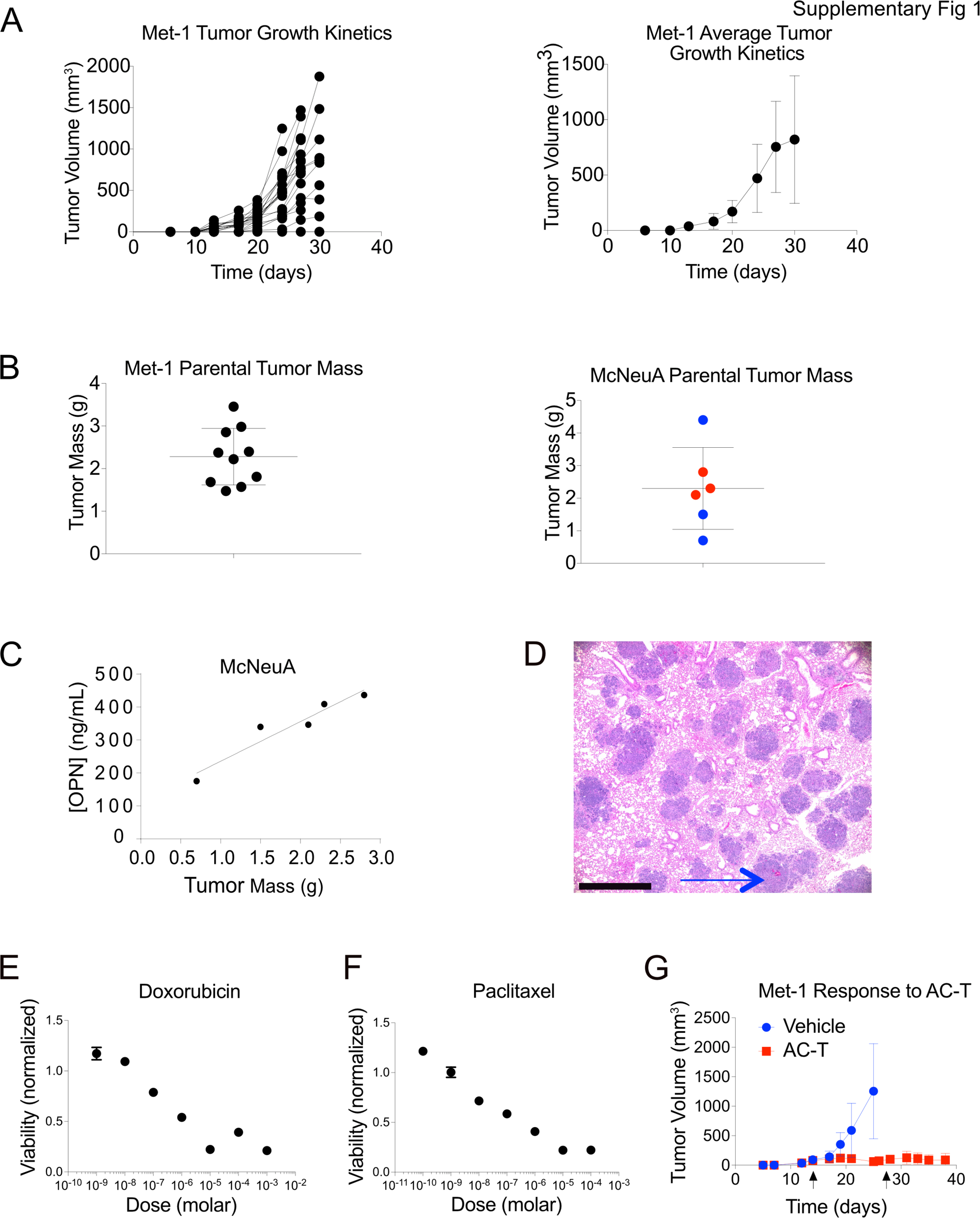

Supplement: S1 Fig — (A) Individual and average tumor growth kinetic rates from FVB mice orthotopically injected with 2.5x10^5^ Met-1 cells. Error bars represent SD. (B) Endpoint tumor masses of mice injected with 105 (red) or 106 (blue) McNeuA cells or 2.5x10^5^ Met-1 cells. Error bars represent SD. (C) Circulating plasma osteopontin (OPN) levels were measured using ELISA and were plotted against the primary tumor mass in the corresponding animal. (D) Representative hematoxylin & eosin staining of lung tissue from a mouse that received intravenous injection of Met-1 cells. An example of a pulmonary metastasis is marked with a blue arrow. Scale = 1000 μm. Representative of two independent experiments. (E,F) Viability of Met1 GFP Luc cells treated in vitro with various doses of doxorubicin and paclitaxel for 72 hours. Representative of three independent experiments. Error bars represent SEM. (G) Tumor growth kinetics of the Met-1 Luc/GFP parental cells injected orthotopically into FVB mice at 2.5 x 10^5^ cells treated with two bi-weekly doses of either vehicle (blue, n = 6) or AC-T (red, n = 8). Error bars represent SEM. (TIF) [file pone.0198790.s001.tif]

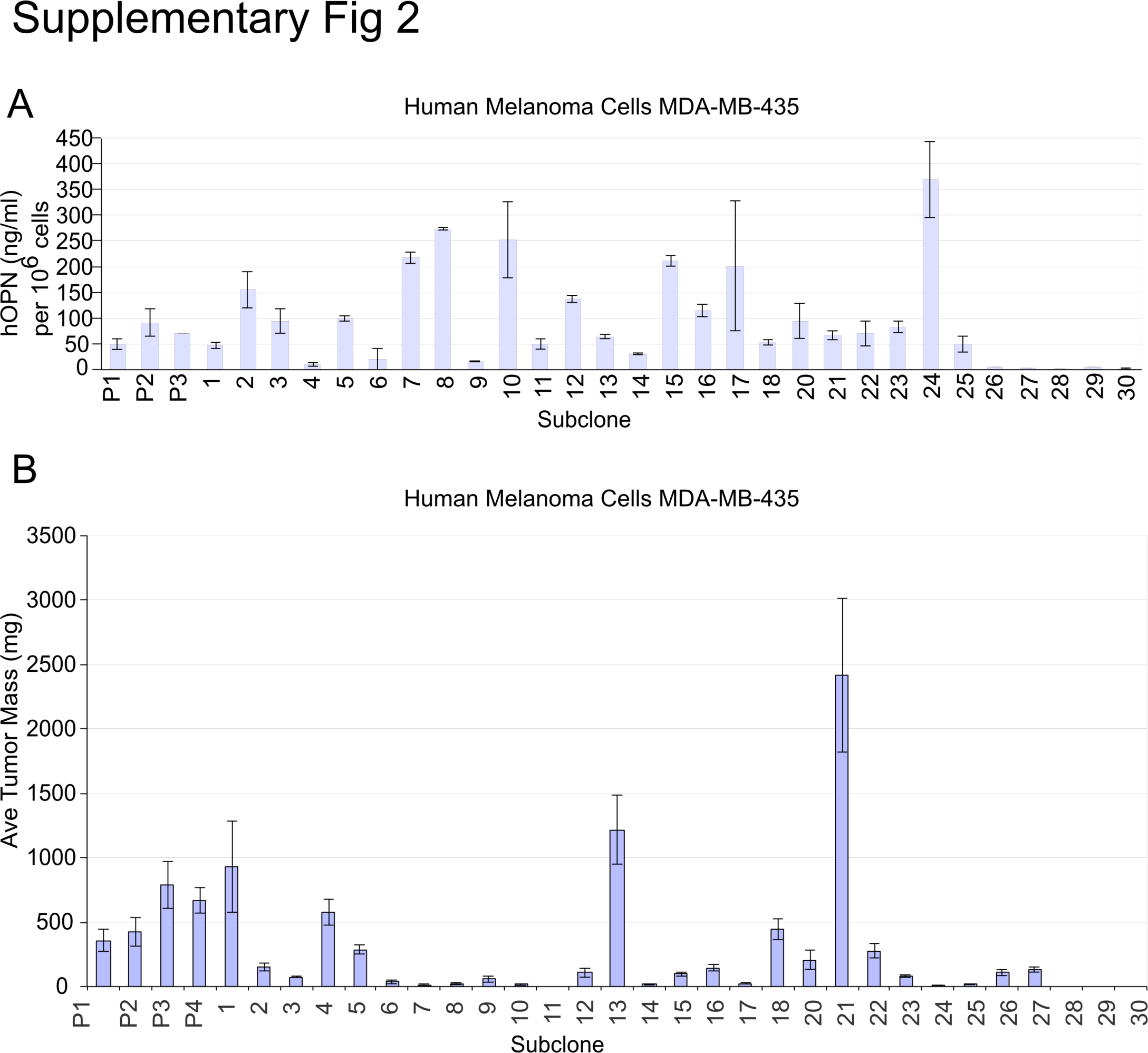

Supplement: S2 Fig — (A) Human osteopontin (hOPN) secreted into culture medium by MDA-MB-435 parental cells (P1-3) and single cell clones after 24h, normalized for the number of cells in each well (n = 3 replicates per cell line). (B) Average mass (mg) of tumors 60 days after subcutaneous injection of 2.5x10^5^ MDA-MB-435 parental cells (P1-4) or indicated subclones into NOD-SCID (n = 5 mice per cohort). (TIF) [file pone.0198790.s002.tif]

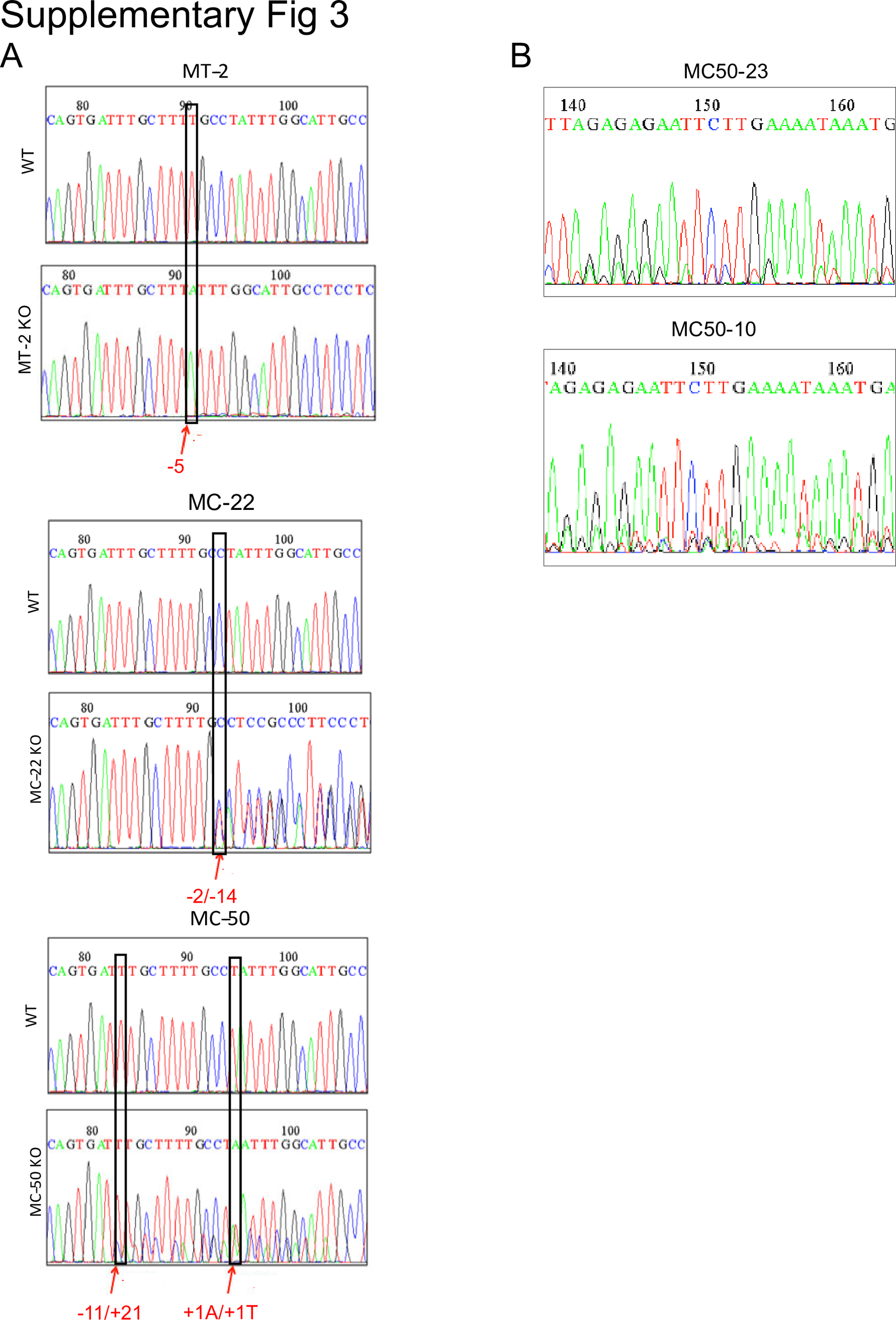

Supplement: S3 Fig — (A) Examples of coding-frame shift confirmed to be homozygous in MT-2, MC-22 and MC-50 clones by Sanger sequencing as reported in Fig 4C. (B) Example of coding-frame shift confirmed to be heterozygous as reported in Fig 4C. (TIF) [file pone.0198790.s003.tif]

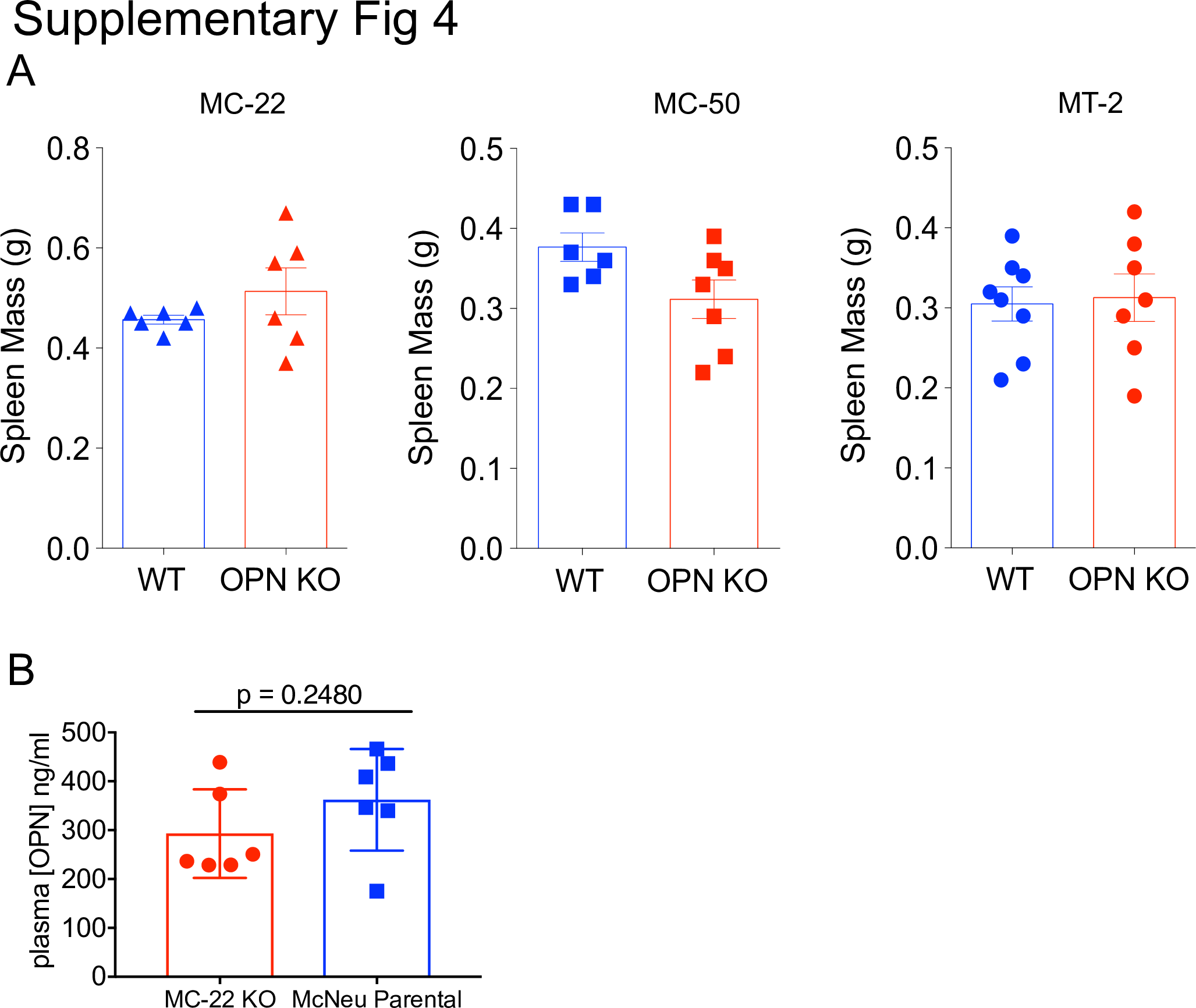

Supplement: S4 Fig — (A) Final spleen mass was measured in mice injected with either MC-22, MC-50, or MT-2 WT or OPN-KO cell lines. No significant difference was observed between WT and KO cohorts for each clone (unpaired, two-tailed Student’s t-test). (B) Circulating plasma mOPN levels were measured from mice bearing either McNeuA Parental or MC-22 OPN-KO primary tumors using ELISA (unpaired, two-tailed t-test, p = 0.2480). Error bars represent SD. (TIF) [file pone.0198790.s004.tif]

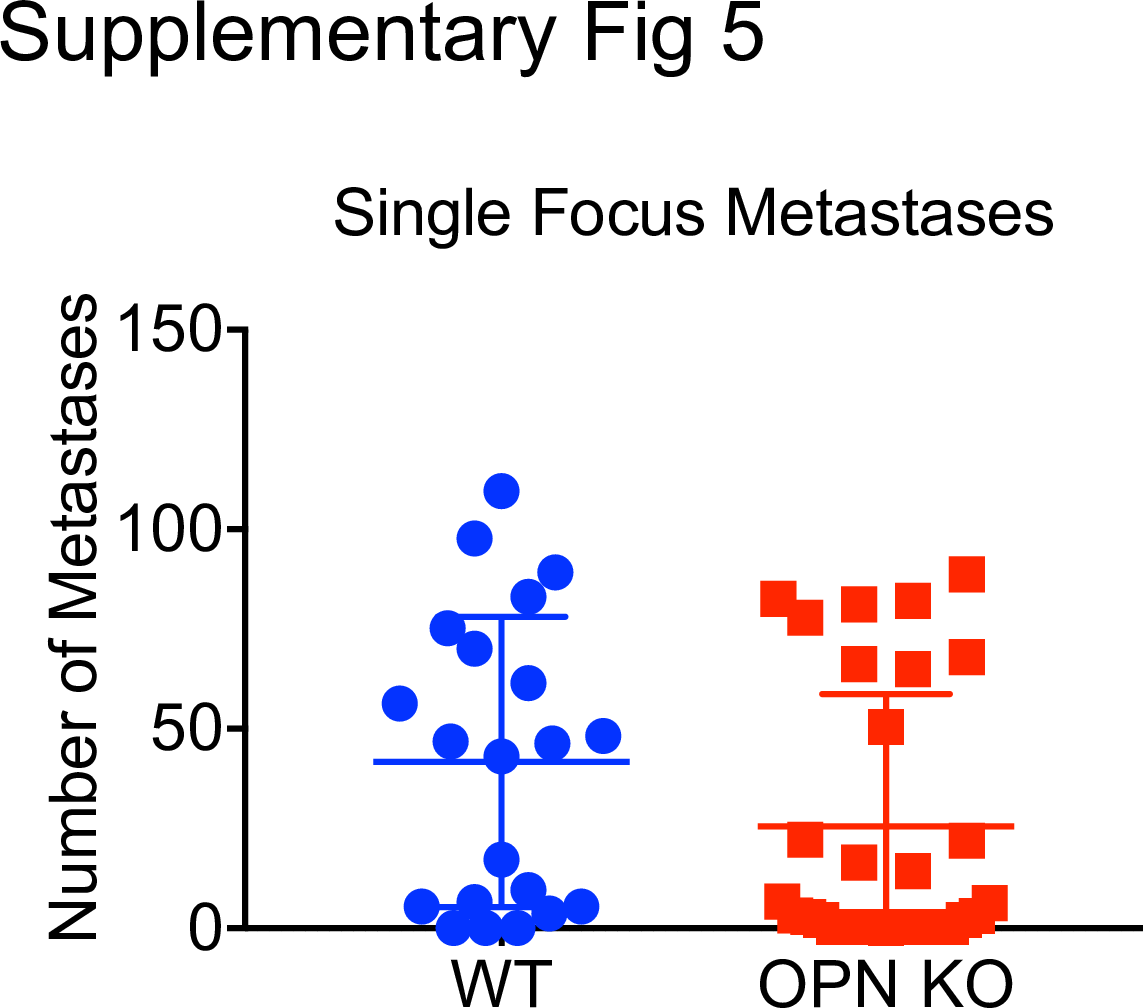

Supplement: S5 Fig — Quantification of single focus metastases in MT-2 WT (blue) and MT-2 OPN KO (red) cohorts (WT n = 21, KO n = 30; Mann-Whitney, p = 0.1248). Error bars represent SD. (TIF) [file pone.0198790.s005.tif]

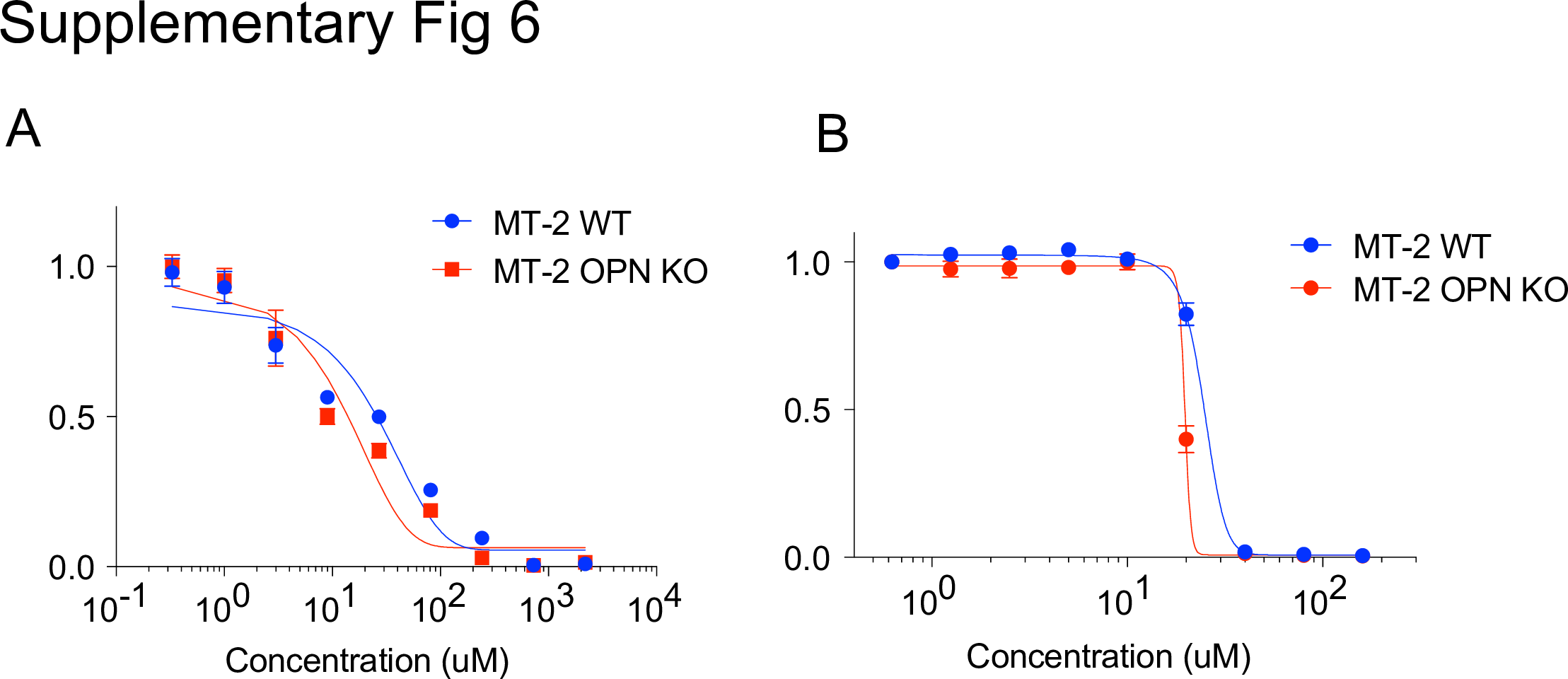

Supplement: S6 Fig — (A,B) MT-2 WT or MT-2 OPN-KO cells were plated in quadruplicate and were treated with various doses of doxorubicin (A) or paclitaxel (B) 24 hours after plating. ATP levels were quantified 72 hours after treatment as a surrogate measure for viability using Cell-Titer Glo and were normalized to vehicle treated. Error bars represent SD. (TIF) [file pone.0198790.s006.tif]
